# Supplementary material for: Plasma Metabolomics Identifies Lipid and Amino Acid Markers of Weight Loss in Patients with Upper Gastrointestinal Cancer
Source: Cancers (Basel). 2019 Oct 19;11(10):1594. doi: 10.3390/cancers11101594 (PMC6826420; doi:10.3390/cancers11101594)
Supplement: Supplementary file 1 [file cancers-11-01594-s001.pdf]

Supplementary Materials: Plasma Metabolomics Identifies Lipid and Amino Acid Markers of Weight Loss in Patients with Upper Gastrointestinal Cancer

Janice Miller, Ahmed Alshehri, Michael I. Ramage, Nathan A. Stephens, Alexander B. Mullen, Marie Boyd, James A. Ross, Stephen J. Wigmore, David G. Watson and Richard J.E. Skipworth

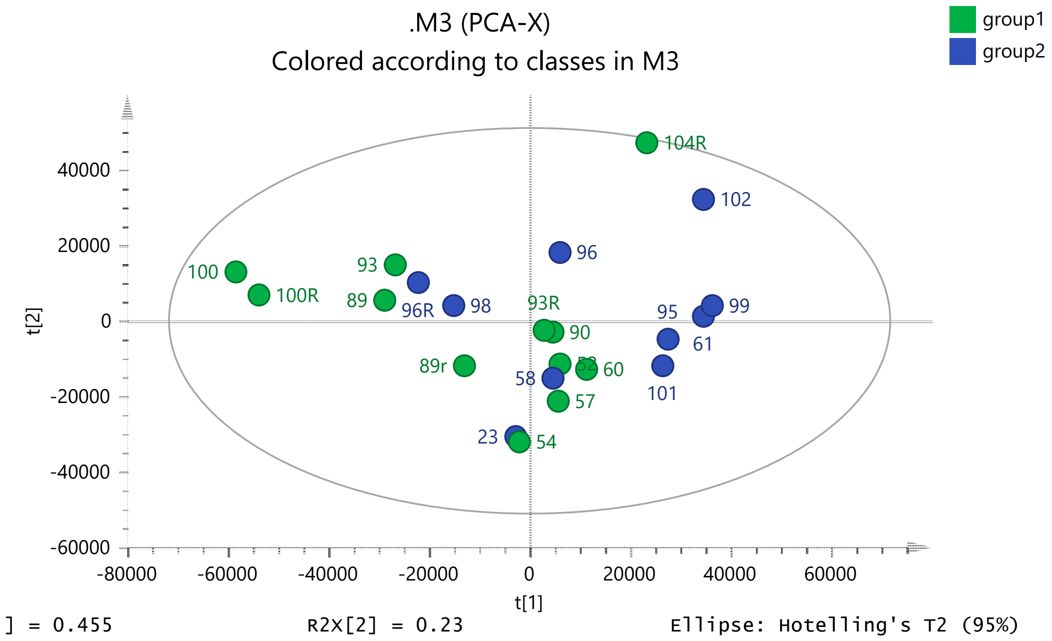

Figure S1. PCA-X analysis. PCA-X analysis of the metabolomics footprint of the 18 plasma samples with pooled samples removed (based on 318 putatively identified metabolites). Green circles (group 1) = WS, blue circles (group 2) = ≥5% WL.

© 2019 by the authors. Licensee MDPI, Basel, Switzerland. This article is an open access article distributed under the terms and conditions of the Creative Commons Attribution (CC BY) license (http://creativecommons.org/licenses/by/4.0/).
